# Wen-Dan Decoction Improves Negative Emotions in Sleep-Deprived Rats by Regulating Orexin-A and Leptin Expression

**DOI:** 10.1155/2014/872547

**Published:** 2014-04-17

**Authors:** Fengzhi Wu, Yuehan Song, Feng Li, Xin He, Jie Ma, Ting Feng, Binghe Guan, Liye Wang, Sinai Li, Xiaolan Liu, Yan Liu, Meng Mao, Jing Liu, Shijing Bai, Cai Song

**Affiliations:** ^1^School of Preclinical Medicine, Beijing University of Chinese Medicine, No. 11 Bei Sanhuan Donglu, Chaoyang District, Beijing 100029, China; ^2^Traditional Chinese Medical Hospital of Ji'nan, Shandong 250012, China; ^3^School of Chinese Medicine, The University of Hong Kong, Pokfulam, Hong Kong; ^4^Graduate Institute of Neural and Cognitive Sciences, China Medical University and CMU Hospital, Taichung 40402, Taiwan; ^5^Research Institute for Marine Drugs and Nutrition, College of Food Science and Technology, Guangdong Ocean University, Zhanjiang 524088, China

## Abstract

Wen-Dan Decoction (WDD), a formula of traditional Chinese medicine, has been clinically used for treating insomnia for approximately 800 years. However, the therapeutic mechanisms of WDD remain unclear. Orexin-A plays a key role in the sleep-wake cycle, while leptin function is opposite to orexin-A. Thus, orexin-A and leptin may be important factors in sleep disorders. In this study, 48 rats were divided into control, model, WDD-treated, and diazepam-treated groups. The model of insomnia was produced by sleep deprivation (SD) for 14 days. The expressions of orexin-A, leptin, and their receptors in blood serum, prefrontal cortex, and hypothalamus were detected by enzyme-linked immunosorbent assay, immunohistochemistry, and real time PCR. Open field tests showed that SD increased both crossing movement (Cm) and rearing-movement (Rm) times. Orexin-A and leptin levels in blood serum increased after SD but decreased in brain compared to the control group. mRNA expressions of orexin receptor 1 and leptin receptor after SD were decreased in the prefrontal cortex but were increased in hypothalamus. WDD treatment normalized the behavior and upregulated orexin-A, leptin, orexin receptor 1 and leptin receptor in brain. The findings suggest that WDD treatment may regulate SD-induced negative emotions by regulating orexin-A and leptin expression.

## 1. Introduction

Insomnia is a common sleep disorder characterized by difficulty falling asleep, staying awake, or both [1]. Worldwide, over 30% of insomnia cases are attributed to high stress or a busy life style [2]. In the USA, about 30–40% of adults have symptoms of insomnia within a given year, and about 10–15% of adults have chronic insomnia [3]. Depression or anxiety, chronic stress, and chronic pain may cause chronic insomnia [4, 5]. Conversely, chronic insomnia may aggravate depression and anxiety due to the dysfunctions of sleep-wake regulating neural circuitries, which have the capacity to reinforce emotional disturbances [6]. The interaction between sleep and emotional changes has been observed both in clinical and subclinical samples, but the molecular mechanisms of the vicious circle are still unclear.

Among many molecular factors, orexin-A and leptin have been associated with insomnia. Orexin-A is a peptide composed of 33 amino acids that have been found in cerebrospinal fluid, lateral and posterior hypothalamus, and medial thalamus [7, 8]. Orexin-A has been shown to play an important role in the sleep-wake cycle and in maintaining the stability of sleep [9–11]. Furthermore, orexin-A administration may strengthen the awake condition and lengthen awake time [12, 13]. The expression of orexin-A increases slowly in dark periods and decreases slowly in periods of illumination. Orexin receptor 1 (OX1R) is activated by orexin-A and is strongly expressed in cerebral cortex and tissue [14, 15].

Leptin is a polypeptide hormone encoded by the obese gene [16]. In contrast to orexin-A, sleep duration increases leptin secretion and sleep deprivation (SD) reduces leptin secretion [17]. The binding of leptin to its receptor (Ob-R) guarantees the function of orexin-A. Furthermore, SD can affect the expression of Ob-R [18]. However, the relationship between orexin-A and leptin in insomnia and in insomnia-induced emotion changes is still unknown. Therefore, exploring the changes in orexin-A, leptin, and the receptors OX1R and Ob-R in sleep-deprived rats is of great importance.

Currently prescribed sleep-aid medications are often associated with many side effects, including excessive drowsiness, impaired thinking, night wandering, agitation, and balance problems. However, Chinese herbal medicines exert more balanced and nourishing effects in the brain, which improve the symptoms of SD in a different way. Wen-Dan Decoction (WDD), a famous extract, has been used clinically by practitioners of traditional Chinese medicine (TCM) with an effective rate of 93.3% [19–22]. Clinical observations indicate that WDD can improve negative emotions to defend against insomnia [23]. However, the molecular mechanism by which WDD improves emotions and sleep is unknown. In the present study, we demonstrate that WDD may improve insomnia-induced negative emotions by regulating orexin-A and leptin expression.

## 2. Materials and Methods

### 2.1. Experimental Animals

Adult male Sprague Dawley rats were purchased from Beijing Vital River Laboratory Animal Technology Limited Company (Beijing, China). Animals were held in a room with a constant temperature of 23 ± 1°C; a relative humidity from 30% to 40%; light for 12 h, from 06:00 to 18:00; darkness for 12 h, from 18:00 to 06:00; and purified water ad libitum. Forty-eight rats were randomly divided into four groups as follows: control group (*n* = 12), model group (*n* = 12), diazepam-treated group (*n* = 12), and WDD-treated group (*n* = 12). The modified multiple platform method was used to generate the SD model rats [24]. Fifteen platforms in a deprivation case (110 cm × 60 cm × 40 cm) were surrounded with water at a temperature between 20°C and 22°C to a depth of 1.0 cm below the surface of the platforms. The rats in the control group were fed routinely for 15 days. The rats in the model, diazepam-treated, and WDD-treated groups were subjected to continuous SD (20 h/day) for 14 days. All animals in the study were maintained in accordance with the guidelines of Chinese legislations on the ethical use and care of laboratory animals. All efforts were made to minimize animal suffering and the number of animals needed to produce reliable data.

### 2.2. Open Field Test

The open field test (OFT) provides a novel environment in which animal locomotion, exploration, and anxiety are measured. The open field arena (100 cm × 100 cm × 40 cm) is constructed of acrylic, with gray walls and a black floor, which is divided into 25 squares of equal areas as previously described [25, 26]. Instances of crossing-movement (Cm) and instances of rearing-movement (Rm) are usually used as measures of exploration and anxiety [27, 28]. A high frequency of these behaviors indicates increased excitability and exploration. Each of the rats was tested for 3 min on experimental day 7 and 14.

### 2.3. WDD Preparation

WDD is comprised of eight different Chinese medicinal herbs (Table 1). The components were purchased from the Pharmaceutical Department of Dongzhimen Hospital, which is affiliated with the Beijing University of Chinese Medicine (BUCM). Director Shihua Gao identified the components, and the voucher specimens were deposited. All of the components were soaked for 1 h at room temperature and decocted with distilled water for 2 h. The filtrates were condensed and dried by a vacuum desiccator at 60°C and then packaged and stored at room temperature for future use.

### 2.4. Drugs and Reagents

Diazepam was purchased from Beijing Yimin Pharmaceutical Co. Ltd. (Beijing, China). Rat orexin-A and leptin enzyme-linked immunosorbent assay (ELISA) kits were obtained from Vector Laboratories, Inc. (Burlingame, CA, USA). The rabbit avidin-biotin-peroxidase complex (ABC) detection kit was obtained from Vector Labs, Inc. (USA) and the rabbit leptin antibody (1 : 200) was obtained from Bioss (Beijing, China). Rabbit orexin-A antibody (1 : 100) was obtained from Millipore Co. (Billerica, MA, USA). Trizol reagent was purchased from Molecular Research Center (Cincinnati, OH, USA). High-Capacity cDNA Reverse Transcription kit was purchased from Applied Biosystems (Foster City, CA, USA). KAPA SYBR FAST qPCR kit was purchased from Kapa Biosystems (Woburn, MA, USA).

### 2.5. ELISA

After the 14-day trial, five rats from each group (control, model, diazepam-treated, and WDD-treated) were anaesthetized with an intraperitoneal injection of 10% chloral hydrate (0.35 to 0.40 mL/100 g body weight). Rats were sacrificed and blood was collected and centrifuged at 3000 rpm for 15 min. The supernatant was collected and stored at −20°C. If precipitate was found during preservation, centrifugation was repeated. Experimental procedures were conducted according to the kit instructions and the concentrations of orexin-A and leptin in serum were detected.

### 2.6. Immunohistochemistry

Expression of orexin-A and leptin in prefrontal cortex and hypothalamus were detected by immunohistochemistry on experimental day 14. Five rats in each group were anesthetized with 10% chloral hydrate (0.35 to 0.40 mL/100 g body weight) and sacrificed. The entire brains were excised and flash-frozen in liquid nitrogen and then moved to −80°C for storage. The brains were dehydrated with 30% sucrose for 24 h and embedded in optimal cutting temperature (OCT) medium after cryoprotection. Frozen brains were cut into slices of 30 *μ*m thickness and fixed with 4% paraformaldehyde for 10 min, then stored at −20°C. A constant-temperature freezing microtome (Leica CM 1900) was used for tissue sectioning. Tissue sections were immersed in 3% H_2_O_2_ to inactivate the endogenous peroxidase and then washed with 0.05 M tris-buffered saline (TBS) three times for 5 min each, blocked by 10% goat serum at room temperature, and incubated with primary antibody at 4°C for over 18 h. After this incubation period, sections were washed with 0.05 M TBS three times for 5 min each, incubated with the secondary antibody at 37°C for 1 h, washed with TBS again, and finally incubated with ABC reagent at 37°C for 1 h. Sections were visualized by DAB for 5 min, dehydrated by graded ethanol solutions, vitrified by dimethylbenzene, and mounted with neutral balsam. Six visual fields were chosen randomly under a microscope at 100x magnification. Quantifications were performed by Image-Pro Plus version 6.0 analysis software to calculate the mean integrated optical density (MOD).

### 2.7. Real-Time PCR

Five rats in each group were randomly selected for analysis of gene expression by quantitative real-time PCR (qPCR). Total RNA from brain tissue was isolated with Trizol reagent according to the manufacturer's protocol. Specifically, total brain tissues in Trizol reagent were homogenized. Mixtures were vortexed in the tube for 15 s and then incubated for 3 min at 20°C. The homogenates were centrifuged at 12,000 ×g for 15 min at 4°C. The supernatants were removed, mixed with diethylpyrocarbonate (DEPC)-treated water (prepared by autoclaving DEPC with water in a ratio of 1 : 1,000), and vortexed vigorously for 15 sec and then centrifuged at 12,000 ×g for 10 min at 4°C. The aqueous layer was collected and added to a one-half volume of 70% ethanol in DEPC-treated water for RNA precipitation. The RNA pellet was collected by centrifugation at 7,500 ×g for 5 min at 4°C and washed twice with 70% ethanol in DEPC-treated water. After air-drying, the RNA pellet was resuspended in 20 *μ*L of DEPC-treated water. Concentrations of extracted RNA were calculated from the UV absorbance at 260 nm. The quality of RNA was assessed by absorbance at 260 nm and 280 nm; 260/280 nm ratios ranging from 1.80 to 2.10 were deemed acceptable. Isolated RNAs were reverse transcribed by the moloney murine leukemia virus (MMLV) reverse transcriptase (RT) with oligo-d(T) primers in a 10 *μ*L reaction using a High-Capacity cDNA Reverse Transcription kit. In detail, 2 *μ*g of total RNA was mixed with 1 *μ*L of 0.5 *μ*g/*μ*L oligo-d(T) primers and RNAase/DNAase-free water in a 10 *μ*L reaction. The mixture was incubated at 70°C for 3 min and then at 37°C for 10 min. The RT reaction mixture of 2 *μ*L of 10x RT buffer, 4 *μ*L of 2.5 mM deoxyribonucleoside triphosphates (dNTPs), 1 *μ*L of enzyme inhibitor, and 1 *μ*L of MMLV RT was incubated at 37°C for 1 min. The two mixtures were combined and incubated at 37°C for 60 min and then at 95°C for 5 min.

Over 40 cycles of qPCR were performed in an Applied Biosystems PCR system with the KAPA SYBR FAST qPCR kit, including SYBR Green Master mix and ROX reference dye, according to the manufacturer's instructions. In brief, complementary DNA (cDNA) was obtained from reverse transcription of the RNA from rat brain. Values were normalized with the use of an internal control (glyceraldehyde 3-phosphate dehydrogenase; GAPDH) in each sample. PCR products were analyzed by gel electrophoresis on a 1.5% agarose gel, and the specificity of amplification was confirmed by the melting curves. Primers employed in qPCR analyses are listed in Table 2.

### 2.8. Statistical Analyses

Data are expressed as mean ± standard error of mean (SEM). One-way analysis of variance (ANOVA) tests were used to analyze the data with statistical package for the social sciences (SPSS) version 17.0 software. In addition, the least significant difference (LSD) method was adopted for comparisons between groups. The repeated measures procedure of the general linear model (GLM) in SPSS version 17.0 was used to conduct one-way ANOVA analysis for repeated measures data (body-weight and food intake), and multivariate analysis process of variance was used to make comparisons between groups on each time point (the LSD method). *P* values <0.05 were considered statistically significant.

## 3. Results

### 3.1. WDD Treatment Ameliorated SD-Induced Increases in Cm and Rm Times

7 days and 14 days after SD, the Cm and Rm times in the model group were significantly increased compared to the control group (*P* < 0.01). However, the Cm times and Rm times in the WDD-treated group were decreased when compared to the model group at 14 days after SD (Cm time: *P* < 0.01; Rm time: *P* < 0.05). Values of both parameters in the WDD-treated group were lower than the corresponding values in the diazepam-treated group (*P* < 0.01) (Table 3).

### 3.2. WDD Inhibited SD-Induced Increase of Orexin-A Content in Blood Serum

Serum levels of orexin-A in the model group were significantly higher than those in the control group (*P* < 0.01), while serum levels of orexin-A in the WDD group were lower than those of the model group (*P* < 0.05) (Figure 5). Leptin serum concentrations in the model group showed no significant differences from those of the control group (*P* > 0.01). (Figure 6).

### 3.3. The Mean Integrated Optical Density (MOD) of Orexin-A and Leptin in Both Prefrontal Cortex and Hypothalamus

**Figure 1:**
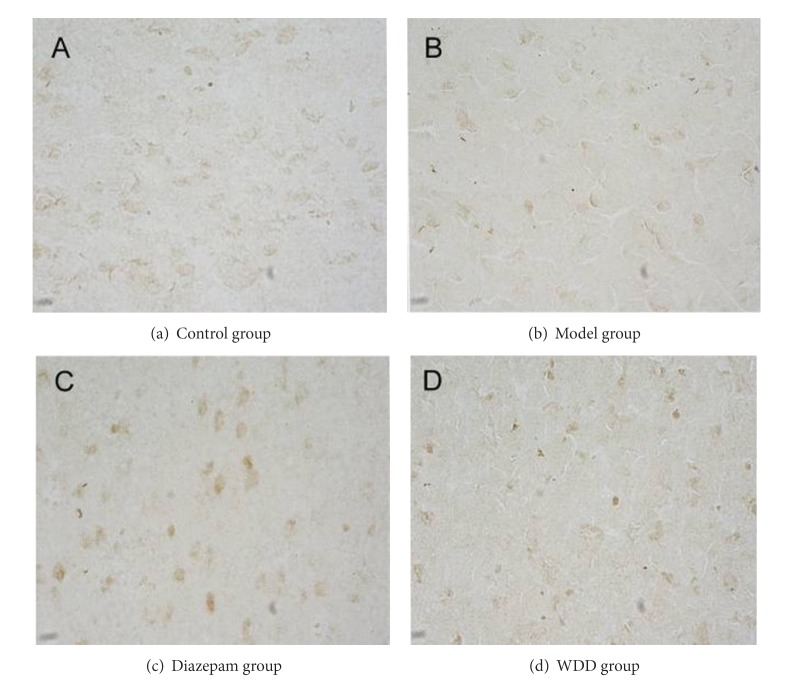
Protein expression of orexin-A in prefrontal cortex. (Tissue sections were viewed at 40x magnification.) Pale brown cells, which are positive for orexin-A expression, are oval-shaped and spread over the endochylema in the prefrontal cortex. No orexin-A positive cells were observed in the cytomembrane.

**Figure 2:**
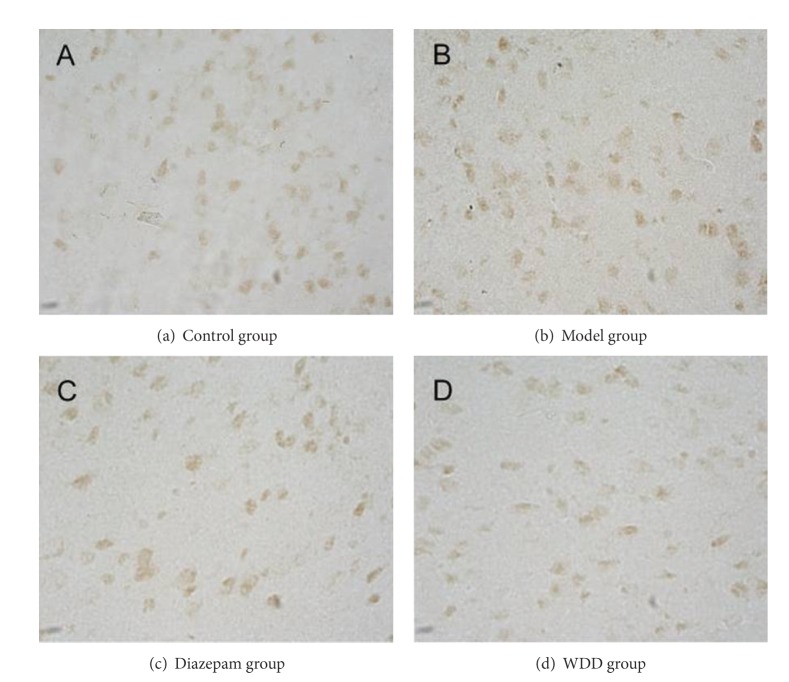
Protein expression of orexin-A in hypothalamus. (Tissue sections were viewed at 40x magnification.) The claybank cells, which are positive for orexin-A expression, are oval-shaped and mainly spread throughout the endochylema in hypothalamus. Fewer positive cells are more apparent in the model group and the diazepam- and WDD-treated groups than the control group.

**Figure 3:**
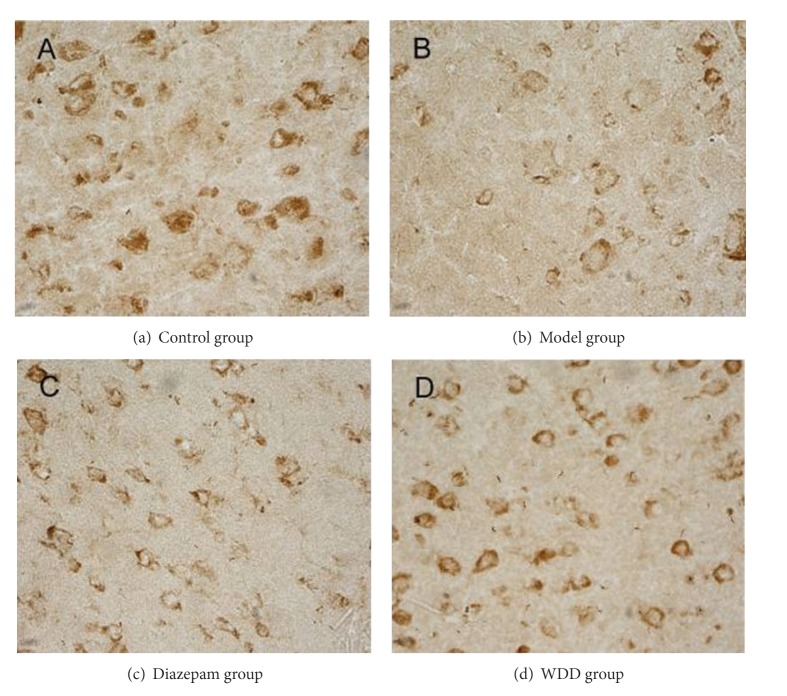
Protein expression of leptin in prefrontal cortex. (Tissue sections were viewed at 40x magnification.) The dark brown cells, which are positive for leptin expression, are circular or oval-shaped and mainly distributed in the cell membrane. Fewer positive cells can be seen in the model group and diazepam-treated groups.

**Figure 4:**
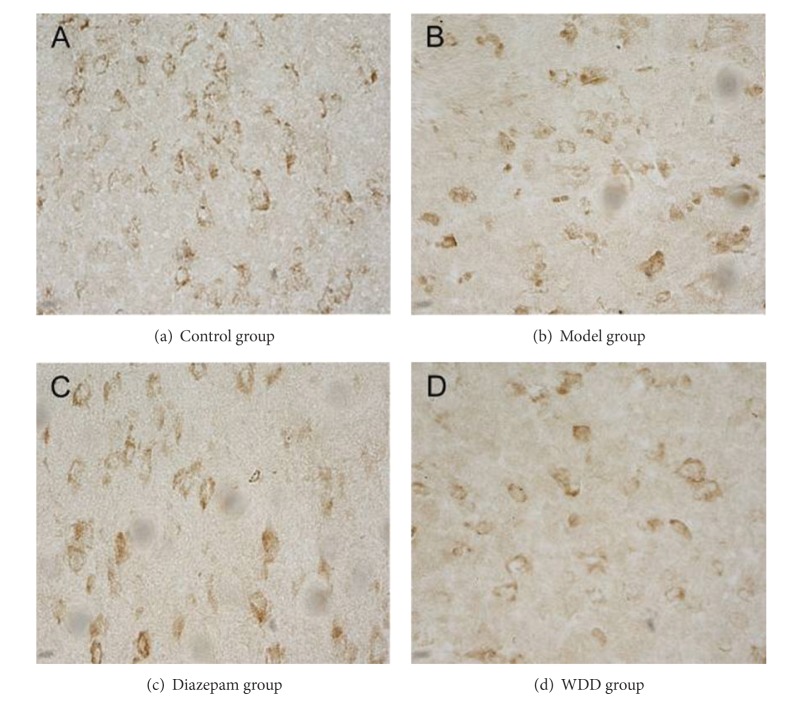
Protein expression of leptin in hypothalamus. (Tissue sections were viewed at 40x magnification.) The dark brown cells, which are positive for leptin expression, are oval-shaped and spread over the cell membrane and the endochylema. Fewer positive cells are apparent in the model group and the diazepam- and WDD-treated groups.

**Figure 5:**
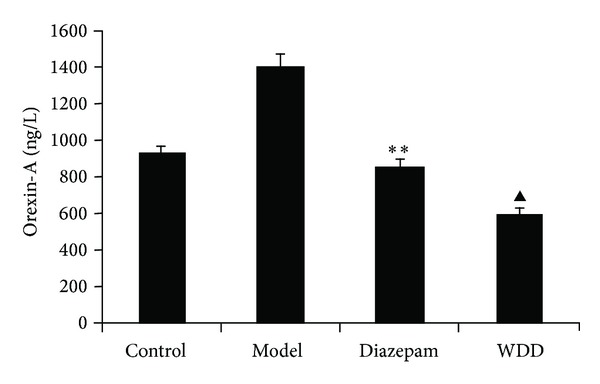
The effect of sleep deprivation on orexin-A concentrations in serum and the regulation of Wen-Dan Decoction. **P < 0.01: versus the control group; ^▲^ *P* < 0.05: versus the model group. Data are presented as mean ± standard error of the mean (SEM).

**Figure 6:**
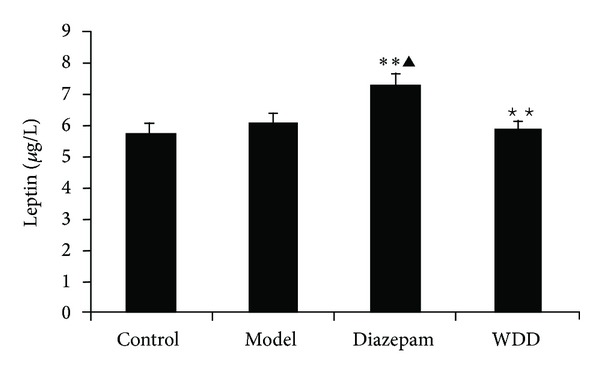
The effect of sleep deprivation on leptin serum concentrations and the regulation of Wen-Dan Decoction. **P < 0.01: versus the control group; ^▲^ *P* < 0.05: versus the model group; ^★★^ *P* < 0.01: versus the diazepam group. Data are presented as mean ± standard error of the mean (SEM).

In both prefrontal cortex and hypothalamus, orexin-A and leptin expressions in the model group were significantly lower than those in the control group (*P* < 0.01) (Figures 1, 2, 3, and 4). Remarkably, the expressions of orexin-A and leptin in the WDD group were significantly higher than those in the model group (*P* < 0.010). Compared to the diazepam group, the expressions of both orexin-A and leptin in the WDD group were significantly higher (*P* < 0.05) (Table 4).

### 3.4. The mRNA Expression of Orexin-A, OX1R, and Ob-R in Prefrontal Cortex and Hypothalamus

Table 5 shows that the mRNA levels of orexin-A in the diazepam- and WDD-treated group were significantly higher than that in the model group in the prefrontal cortex (*P* < 0.01). Regarding the mRNA level of OX1R in the prefrontal cortex, the diazepam- and WDD-treated groups showed increased expressions when compared to the model group (*P* < 0.05). Compared to the control group, the mRNA levels of Ob-R in the model group in both prefrontal cortex and hypothalamus were significantly increased (*P* < 0.01).

## 4. Discussion

The present study showed that SD significantly increased Cm and Rm time in rats in the open field, while WDD treatment reversed these changes. Especially, at 14 days after SD, the Rm time in the WDD-treated group was significantly lower than that in the diazepam-treated group (*P* < 0.01). Although the anxiolytic effects of sleep deprivation on OFT results has been demonstrated in a previous study [29], the paradigm used previously (72 h total) differed from ours (20 h/day for 14 days). In an OFT, many factors, such as breeding environment, sex, and stress reaction to water and food deprivation, may affect the results [30]. Diazepam is popularly used to treat insomnia; however, in the present study, the behavioral measurements did not show any beneficial effect after SD in both Cm time and Rm time in the diazepam-treated group. Similar results were reported by others [31], which may indicate that diazepam works through other behavioral aspects, such as stretch attend and wall-following (thigmotaxis) [31], rather than the locomotor and exploring activities, as observed in the OFT.

After long-term SD, significantly decreased protein expression of orexin-A was observed in prefrontal cortex and hypothalamus, but mRNA expression of orexin-A in prefrontal cortex and hypothalamus of the model group was lower than the control group, though this latter difference did not reach statistical significance. A previous study [32] showed that long-term (48 h) SD downregulated the positive expression of orexin-A, decreased OX1R mRNA expression in the prefrontal cortex, and increased OX1R mRNA expression in the hypothalamus. Because orexin-A can maintain the awake state, our results indicate that long-term SD (≥14 d) can affect the rat's ability to wake up, as well as vigilance, which may be related to reductions in orexin-A expression induced by high consumption of glucose and restrained protein synthesis. On the other hand, SD decreased the expression of leptin in the serum, prefrontal cortex, and hypothalamus and decreased mRNA expression of Ob-R in prefrontal cortex. Previously, leptin expressions have been reported to decline after SD in the serum of young rats [32]. The mechanism of leptin may be mediated by its binding with Ob-R in the central nervous system and peripheral tissues. Acute illumination for 4 h did not change orexin expression in lateral hypothalamus, while SD for 6 h significantly increased orexin expression [33–35]. These results demonstrate that SD may induce the dysfunction of this pair of molecules.

As mentioned above, WDD is a famous Chinese medicine that has been prescribed for hundreds of years and that has a remarkable effect in the treatment of insomnia.

It is read that “Ban xia, Zhu ru, Zhi shi 2* liang* each, Chen pi 3* liang*, Zhi gancao 1* liang*, Fu ling 1.5* liang*, Sheng jiang 5 pieces, and Chinese date, 1 piece.” in* Treatise on Three Categories of Pathogenic Factors* written by Chen Yan [36] in Song dynasty. Phlegm-fire disturbing heart and the heart spirit restless cause insomnia was written in* Jing Yue Quan Shu Bu Mei*. Chinese clinical literature shows that WDD is effective in the treatment of insomnia. Researchers [19] conducted a clinical trial with 150 insomnia patients, using WDD as a treatment medication and Ambien as a control medicine. The results showed that, compared to the control group, sleep time, sleep efficacy, sleep disorder, and daytime activity function of the treatment group were significantly improved.

Results from the present study show that the excitability behavior of rats is enhanced on day 14 after SD. Improvements were observed after both WDD and diazepam treatments, and WDD effects were superior to those of diazepam. WDD does not function as a sedative-hypnotic; rather, WDD regulates the emotional disorders caused by insomnia, thus improving sleep quality. The observed enhanced excitability caused by SD in our study is in accordance with other researchers' findings [37, 38]. WDD can significantly improve rat excitability behavior caused by SD on day 14 after SD. The beneficial effects of WDD are also superior to diazepam. After 14 days of SD, WDD treatment decreases Cm and Rm time, indicating that WDD can improve SD-induced excitability behavior. As well, WDD significantly improves the protein expression of orexin-A in the serum, the prefrontal cortex, and hypothalamus, though no statistically significant improvements in mRNA expression of orexin-A and OX1R in the hypothalamus were observed, which indicates that WDD may regulate orexin-A at the protein, rather than mRNA, level. Even though WDD could not improve Ob-R expression in the prefrontal cortex, Ob-R expression in the hypothalamus was remarkably increased. Considering that Ob-R is located mainly in the hypothalamus, the results indicate that the hypothalamus is the key target of leptin. Furthermore, WDD treatment increased leptin and promoted its binding to Ob-R, inhibiting the synthesis and secretion of orexin-A, a hypothalamic neuropeptide that may enhance sympathetic nervous system activity, decrease appetite, and promote energy consumption. As a result, the emotional changes induced by SD are changed. Protein expressions of orexin-A in the prefrontal cortex and hypothalamus, as well as mRNA expression of orexin-A and OX1R in the prefrontal cortex, increased significantly, which demonstrates that WDD can activate OX1R by inducing the secretion and expression of orexin-A in the brain, thus reducing glucose consumption and promoting protein synthesis. In a similar manner, diazepam also elevated the protein level of leptin in the serum and hypothalamus. However, WDD is superior to diazepam, with the advantage of multitargeted regulation, as well as the improvement of negative emotions.

## 5. Conclusion

In conclusion, the present study demonstrated that SD is a good model to induce insomnia-related negative emotions. WDD treatment effectively improved SD-induced negative emotions by regulating the function of orexin-A and leptin, such as the upregulated orexin-A and leptin in blood serum and brain tissue of SD rats compared to control animals. Furthermore, our study demonstrated that WDD can upregulate orexin-A, OX1R, and Ob-R in the prefrontal cortex of the SD rats, while only upregulating Ob-R in hypothalamus. WDD treatment has been proven effective in improving SD-induced negative emotions by regulating orexin-A and leptin, an effect that may have a great impact on the treatment of patients with insomnia.

## Figures and Tables

**Table 1:** Composition and active compounds of WDD.

Components	Voucher specimens number	Part used	Active Compounds	Amount used (g)
*Pinelliaternata *	3002305058	Tuber	Total alkaloids	6
Immature bitter orange	100381601	Young fruit	Flavones	6
*Citrus reticulate* Blanco	100580191	Mature pericarp	Hesperidin, citrus flavonoids	9
Bamboo shavings	100382441	Interlayer of stem	Phosphodiesterase inhibitor	6
Liquorice	100480341	Rhizome	Triterpenoid saponins	3
Ginger	100186553	Rhizome	Gingerol	5
Poriacocos	100382861	Sclerotium	Polysaccharides	4.5
Fructus ziziphi jujubae	100118527	Fruits	Alkaloid and glycoside	5

**Table 2:** Primer sequences, length of PCR products, and optimal annealing temperature for each gene used in real-time quantitative PCR.

Primer	Sequence (5′-3′)	*T*_*a*_ (°C)	bp
GAPDH	F: 5′GGAAAGCTGTGGCGTGAT3′ R: 5′AAGGTGGAAGAATGGGAGTT3′	60	308

Orexin-A	F: 5′CGCCAGAAGACGTGTTCCT3′ R: 5′GCCGCTTTCCCAGAGTGAG3′	60	88

Orexin receptor 1	F: 5′TTTCGGGAGCAGTTCAAGG3′ R: 5′CCCCAGGCAAAGGATCAA3′	60	203

OB-R	F: 5′GCAGTCCAGCCTACACTCTTG3′ R: 5′GCTTCACCACATACCTCCTCAC3′	60	171

**Table 3:** WDD treatment ameliorated SD-induced increases in Cm and Rm times.

Parameters	Groups	Before SD	7 days after SD	14 days after SD
Cm times	Control group	40.9 ± 19.7	31.3 ± 23.1	8.8 ± 4.4
	Model group	50.2 ± 11.4	89.0 ± 21.3**	53.3 ± 15.6**
	Diazepam group	39.4 ± 16.5	105.4 ± 15.0**	44.4 ± 22.8**
	WDD group	45.7 ± 23.8	96.6 ± 35.7**	25.0 ± 12.7^∗▲★★^

Rm times	Control group	7.7 ± 4.4	2.1 ± 1.8	1.0 ± 1.0
	Model group	6.4 ± 2.2	9.8 ± 2.5**	4.0 ± 2.1**
	Diazepam group	5.3 ± 3.5	10.6 ± 4.8**	4.3 ± 1.2**
	WDD group	6.8 ± 3.7	11.4 ± 2.5**	2.0 ± 0.9^∗▲^

*P < 0.05, **P < 0.01: versus the control group; ^▲^ *P* < 0.05: versus the model group; ^★★^ *P* < 0.01: versus the diazepam group.

**Table 4:** The effect of sleep deprivation on the mean integrated optical density (MOD) of orexin-A and leptin in the prefrontal cortex and hypothalamus and the regulation of WDD.

Part	Groups	Orexin-A	Leptin
Prefrontal cortex	Control group	0.17 ± 0.03	0.40 ± 0.02
	Model group	0.14 ± 0.02**	0.19 ± 0.04**
	Diazepam group	0.14 ± 0.01**	0.22 ± 0.03^∗∗▲^
	Wen-dan group	0.16 ± 0.01^▲▲∗∗^	0.27 ± 0.03^∗∗▲▲^

Hypothalamus	Control group	0.17 ± 0.01	0.27 ± 0.03
	Model group	0.12 ± 0.02**	0.15 ± 0.04**
	Diazepam group	0.13 ± 0.02**	0.18 ± 0.03^∗∗▲▲^
	Wen-dan group	0.14 ± 0.01^∗∗▲▲★^	0.21 ± 0.02^∗∗▲▲★★^

**P < 0.01: versus the control group; ^▲^ *P* < 0.05: versus the model group; ^▲▲^ *P* < 0.01: versus the model group; ^★^ *P* < 0.05: versus the diazepam group; ^★★^ *P* < 0.01: versus the diazepam group. Data is presented as mean ± standard error of the mean (SEM).

**Table 5:** The effect of sleep deprivation on mRNA expression of orexin-A, OX1R, and Ob-R in prefrontal cortex and hypothalamus and the regulation of WDD.

Part	Group	Orexin-A	OX1R	OB-R
Prefrontal cortex	Control group	1.62 ± 0.54	0.80 ± 0.34	1.10 ± 0.10
	Model group	0.70 ± 0.20	0.34 ± 0.14	0.61 ± 0.13**
	Diazepam group	1.65 ± 0.22^▲▲^	0.64 ± 0.11^▲^	1.91 ± 0.07^∗∗▲▲^
	Wen-dan group	2.15 ± 0.04^▲▲★^	0.79 ± 0.10^▲^	0.79 ± 0.07^∗★★^

Hypothalamus	Control group	2.41 ± 1.41	0.82 ± 0.23	1.28 ± 0.45
	Model group	1.91 ± 1.01	0.99 ± 0.04	2.55 ± 0.23**
	Diazepam group	1.22 ± 0.34	0.78 ± 0.26	2.71 ± 0.24^∗∗▲^
	Wen-dan group	2.09 ± 0.17	1.65 ± 0.74	4.30 ± 0.76^∗▲★^

*P < 0.05: versus the control group; **P < 0.01: versus the control group; ^▲^ *P* < 0.05: versus the model group; ^▲▲^ *P* < 0.01: versus the model group; ^★^ *P* < 0.05: versus the diazepam group; and ^★★^ *P* < 0.01: versus the diazepam group. Data are presented as mean ± standard error of the mean (SEM).
